# Spatial variation of yield response and fertilizer requirements on regional scale for irrigated rice in China

**DOI:** 10.1038/s41598-019-40367-2

**Published:** 2019-03-05

**Authors:** Xinpeng Xu, Ping He, Mirasol F. Pampolino, Shaojun Qiu, Shicheng Zhao, Wei Zhou

**Affiliations:** 1 Institute of Agricultural Resources and Regional Planning, Chinese Academy of Agricultural Sciences (CAAS), Beijing, 100081 P.R. China; 2 International Plant Nutrition Institute (IPNI) China Program, CAAS-IPNI Joint Lab for Plant Nutrition Innovation Research, Beijing, 100081 PR China; 3 International Plant Nutrition Institute (IPNI), Southeast Asia Program, c/o IRRI, Los Baños, Laguna, Philippines

## Abstract

A large number of on-farm experiments (*n* = 5556) were collected for the period 2000–2015 from the major rice (*Oryza sativa* L.) producing regions in China, to study the spatial variability of attainable yield, yield response, relative yield and fertilizer requirements at regional scale, by coupling geographical information system with the *Nutrient Expert for Rice* decision support system. Results indicated that average attainable yield was 8.8 t ha^−1^ across all sites, with 18.3% variation. There were large variations in yield response to nitrogen (N), phosphorus (P), and potassium (K) fertilizer application with coefficients of variation of 39.2%, 57.0%, and 53.4%, and the sites of 73.4%, 85.8%, and 87.6% in the study area ranged from 2.0 to 3.0, from 0.7 to 1.3, and from 0.7 to 1.3 t ha^−1^, respectively. Mapping the spatial variability of relative yield to N, P, and K indicated that the sites of 78.6%, 92.4%, and 88.7% in the study area ranged from 0.65 to 0.75, from 0.80 to 0.92, and from 0.84 to 0.92, respectively. The high yield response and low relative yield to N and P were mainly located in the Northeast (NE), Northwest (NW), and north of the Middle and Lower Reaches of Yangtze River (MLYR) regions. The spatial distribution of N, P, and K fertilizer requirements ranged 140–160 kg N ha^−1^, 50–70 kg P_2_O_5_ ha^−1^ and 35–65 kg K_2_O ha^−1^ which accounted for 66.4%, 85.5% and 73.0% of sites in the study area, respectively. This study analyzed the spatial heterogeneity of attainable yield, soil nutrient supply capacity and nutrient requirements based on a large database at regional or national scale by means of geographical information systems and fertilizer recommendation systems, which provided a useful tool to manage natural resources, increase efficiency and productivity, and minimize environmental risk.

## Introduction

Rice (*Oryza sativa* L.) yield has greatly increased in the past two decades and plays a vital role in guaranteeing food security and promoting economic development in China. The rice area harvested and production in China accounted for 19.1% and 28.5%, respectively, of the worlds’ total^1^; the equilibrium of rice supply and demand has great impact on the stability of the world grain market. However, there are unprecedented pressures on the current agricultural and natural resources to meet the increasing food demand. The problems of resource shortages and environmental pollution bring great challenges to sustainable development of agricultural production in China^2,3^.

The rice yield has increased continuously to meet population growth while the area of arable land has decreased, because of variety improvement, fertilizer application, and optimization of cultivation techniques and management measures^4–6^. However, low fertilizer use efficiency is an outstanding and serious problem in current rice production. Studies showed that nitrogen (N) use efficiency was less than 30%, and phosphorus (P) recovery efficiency was below 20% for most famers’ fertilization practices in many regions of China^7,8^. Low nutrient use efficiency means that more nutrients accumulated in the soil or were lost to the environment. Chen *et al*.^3^ study showed that the N surplus was 46–280 kg N ha^−1^ in farmers’ fields, and the Olsen P concentration in the top layer (0–20 cm) has exceeded 20 mg kg^−1^ in many regions for rice systems in China^9^. These high nutrient concentrations are also implicated in the substantial greenhouse gas emissions and serious water eutrophication in rivers and lakes^10,11^.

As an important component of agricultural production, optimized or balanced fertilization can improve crop yields, save resources and protect the environment. Many methods have been adopted to optimize fertilization rates and improve nutrient management to increase nutrient use efficiency and reduce environmental pollution. These include: fertilizer requirements based on the interactions of indigenous nutrient supply, yield target and crop nutrient demand^12^, fertilizer management according to alternate wetting and drying irrigation^13^, optimizing nutrient management strategies in combination with leaf color chart and optimum fertilizer placement^14^, site-specific nutrient management with water management and optimized transplanting density^15^. These methods have potentially increased rice yield by 10% and nutrient use efficiency by 30% over farmers’ practices.

At present, fertilizer recommendations based on individual or few data cannot meet the demand of the current intensive agricultural production, but the spatial variability in climate, soil fertility and management practices must be considered when developing the most cost efficient nutrient management strategy. However, it is difficult to develop a variable rate technique by studying the spatial variability of soil nutrients in the family responsibility system in China and to communicate the great need to establish a fertilizer recommendation method based on crop responses^16^. Geostatistics has been adopted to assess spatial variability of grain yield and nutrient balance at regional scale to develop reasonable nutrient management^17,18^, which will contribute to realize the rational allocation and efficient use of fertilizer resources^19^.

Rice production must increase to meet future food requirements amid strong competition for limited resources and environmental pollution. Understanding the distribution of attainable yield, yield response to fertilizer application and soil nutrient supply capacity are necessary in developing fertilizer recommendations, which will help to reform agricultural policies aimed at the characteristics of agricultural production in China. Therefore, we combined the *Nutrient Expert for Rice* system and geostatistical analysis to (1) analyze the current rice attainable yield distribution; (2) map the distribution of yield response and relative yield to fertilizer application; and (3) explore spatial variation of fertilizer requirements at regional scale.

## Materials and Methods

### Study area and data source

**Figure 1:**
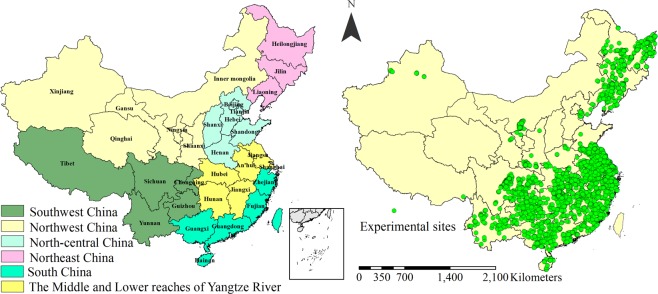
Geographical distribution of the studied locations and rice experiment sites in the six regions of China.

The database used in this study was obtained from 5,556 field experiments conducted by the International Plant Nutrient Institute China Program and the results of these studies were published in scientific journals from 2000 to 2015, which covered all the main rice-producing regions including several distinct agroecosystems in China (Fig. 1). In the Wed of science, the following keywords were used to search the literature: rice, yield, nutrient use efficiency, nutrient uptake. The data in the articles must be from filed experiment under carefully controlled conditions, and harvest index less than 0.4 was excluded in building Nutrient Expert system because these data were treated as crop suffering stress from water, biotic or abiotic stress^20^. The four rice types in terms of season (early, middle, late and single-season rice) were farmed using intensive cultivation methods. The experiments included optimum fertilization experiments, long-term field experiments, and different fertilizer application rates, varieties, water and fertilizer management across six regions of China. Single-season rice is mainly cultivated in a mono-rice system in northeast (NE) and northwest China (NW) where a cool-temperate climate is dominant, and is grown from early or middle May to middle or late September. Middle rice is mainly cultivated in the middle and lower reaches of the Yangtze River (MLYR, such as Hubei, An’hui, Jiangsu and Zhejiang provinces), southwest (SW), and some provinces in north-central China (NC, Henan and Shandong provinces). These regions are dominated by temperature and subtropical climates, and rice crops are rotated with winter wheat, rape, or other crops. The rice crops in these regions are grown from late May or early June to late September or early October. Early and late rice are mainly concentrated in south China (SC) and in some southern provinces of the MLYR (Jiangxi, Hunan, and south of Hubei and Zhejiang provinces) that have a subtropical and tropical climate, where they are grown in an early/late rice rotation system, early rice is grown from late March or early April to early or middle July, late rice is grown from late July or early August to late October or middle November.

### Nutrient Expert for Rice decision support system

The *Nutrient Expert for Rice* decision support system was used to recommend fertilizer rate for each experimental site in the current study. The principle of the method is based on agronomic efficiency (yield increase per unit of fertilizer) and yield response, which combine with the Quantitative Evaluation of the Fertility of Tropical Soils (QUEFTS) model^20,21^ and “4R” principles (applying the right source of nutrient at the right rate and the right time in the right place) to determine fertilizer rate and develop nutrient management practices. Agronomic efficiency is related to yield response and fertilizer application rate, and determined by optimal amounts of added nutrients to provide a reasonable value. The relationships among yield response, agronomic efficiency, relative yield and soil indigenous nutrient supply were built to support fertilizer recommendation. The N recommendation was based on yield response to fertilizer and agronomic efficiency of applied N. While P and potassium (K) requirements considered target yield, yield response, nutrient balances and residual nutrients from the previous crop. The fertilization principle and parameterization of the Nutrient Expert system have been described in detail and reported by Pampolino *et al*.^22^ and Xu *et al*.^16,23^. Nutrient Expert system combines all the steps and guidelines in site-specific nutrient management into simple software, and gives a dynamic fertilizer management that are tailored to the specific field due to different environmental conditions, management practices from current and previous residual nutrient, in order to take full advantage of soil nutrient. In the current study, the system assumed that straw was not returned into soil for single-season rice because of low temperature, while all straw was incorporated into soil after harvest for early, middle and late rice planting regions.

### Analysis

An optical remote sensing image based on terrestrial ecosystems was obtained to identify the rice fields from land cover classification. The software of GS + 5.3 (Plainwell, Michigan, USA) and ArcGIS 9.3 software (ESRI, Redlands, USA) were adopted to map the distribution of attainable yield, yield response, relative yield and fertilizer requirements across the study plots using semivariogram models in conjunction with kriging interpolation. For the early-late rice rotation system and the multiple-year experiments, the average value was adopted at the same site. In this study, attainable yield is the maximum yield derived in field experiments, which is determined from field trials or local experts’ experience for a geographic region or growing environment according to site characteristics and farmers’ actual yield; yield response to fertilizer N, P, and K is the yield gap between full NPK plots and omission plots in which one of the nutrients is omitted, yield response is determined by attainable yield and soil fertility and inversely related to the soil fertility, the higher soil fertility, the lower yield response; relative yield is defined as the ratio between nutrient-limited yield from the nutrient omission plot and attainable yield from the ample NPK plot, the percentile of relative yield was used to estimate yield response when yield response data are unavailable^22^; fertilizer N, P_2_O_5_, and K_2_O requirements were calculated based on *Nutrient Expert for Rice*^23^.

$${\rm{Fertilizer}}\,{\rm{N}}={{\rm{YR}}}_{{\rm{N}}}/{{\rm{AE}}}_{{\rm{N}}}$$

$${\rm{Fertilizer}}\,{\rm{P}}=({{\rm{YR}}}_{{\rm{P}}}\times {{\rm{RIE}}}_{{\rm{P}}}/{{\rm{RE}}}_{{\rm{P}}}+{\rm{Ya}}\times {{\rm{RIE}}}_{{\rm{P}}}\times {{\rm{HI}}}_{{\rm{P}}}\times {{\rm{X}}}_{{\rm{G}}} \% )\times 2.292$$

$$\begin{array}{lll}{\rm{F}}{\rm{e}}{\rm{r}}{\rm{t}}{\rm{i}}{\rm{l}}{\rm{i}}{\rm{z}}{\rm{e}}{\rm{r}}\,{\rm{K}} & = & ({{\rm{Y}}{\rm{R}}}_{{\rm{K}}}\times {{\rm{R}}{\rm{I}}{\rm{E}}}_{{\rm{K}}}/{{\rm{R}}{\rm{E}}}_{{\rm{K}}}+{\rm{Y}}{\rm{a}}\times ({{\rm{R}}{\rm{I}}{\rm{E}}}_{{\rm{K}}}\times {{\rm{H}}{\rm{I}}}_{{\rm{K}}}\times 100{\rm{ \% }}+{{\rm{R}}{\rm{I}}{\rm{E}}}_{{\rm{K}}}\\  &  & \times (1-{{\rm{H}}{\rm{I}}}_{{\rm{K}}})\times {{\rm{X}}}_{{\rm{S}}}{\rm{ \% }}))\times 1.205\end{array}$$

Where the unit of fertilizer N, P, and K are fertilizer N, P_2_O_5_, and K_2_O requirements (kg ha^−1^), respectively, YR is yield response (kg ha^−1^), AE is agronomic efficiency (kg kg^−1^), RIE is nutrient uptake requirement per ton of grain yield (kg ha^−1^), RE is recovery efficiency to nutrient application (%), Ya is attainable yield (kg ha^−1^), HI is harvest index, X_G_% and X_S_% are the nutrient return proportion of grain and straw, respectively, and 2.292 and 1.205 are conversion constant for P and K, respectively.

## Results

### Distribution of attainable yield

**Figure 2:**
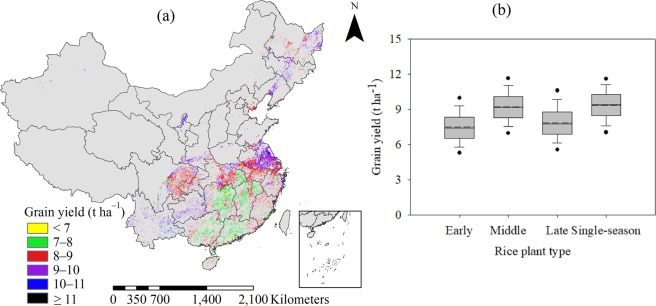
Distribution of rice attainable yield (**a**) in China, and the box plots of grain yield for four rice planting types in this study (**b**), solid and dashed lines indicate median and mean, respectively. The box boundaries indicate the upper and lower quartiles, the whisker caps indicate 90th and 10th percentiles, and the circles indicate the 95th and 5th percentiles.

The attainable yield showed an obvious spatial distribution in regions and plant types. The attainable yield in middle and single-season rice planting regions was higher than in early and late rice planting regions (Fig. 2a). On average, attainable yield was 8.8 t ha^−1^ across all sites, with 18.3% variation. The attainable yield values were 7.5, 9.3, 7.9, and 9.4 t ha^−1^ for early, middle, late and single-season rice, with 18.6%, 15.8%, 18.9%, and 15.1% variation, respectively (Fig. 2b). Sites with attainable yield of <8 t ha^−1^ accounted for 22.4% of the study area and were mainly distributed in double-season rice planting regions, such as the south MLYR and SC; of these, 1.7% of sites in the study area had attainable yield of <7 t ha^−1^. Sites with attainable yield in the range of 8 to 9 t ha^−1^ accounted for 42.0% of the study area, and were mainly distributed in the central MLYR, north SW and northwest NE, and east SC. Sites with attainable yield in the range of 9 to 10 t ha^−1^ accounted for 30.3% of the study area, and were mostly in the north MLYR, and SW and NE regions. We found that 5.3% of sites in the study area exceeded 10 t ha^−1^ across all regions, mainly distributed in the southeast SW, central NW and south NE regions.

### Spatial distribution of yield response and relative yield

**Figure 3:**
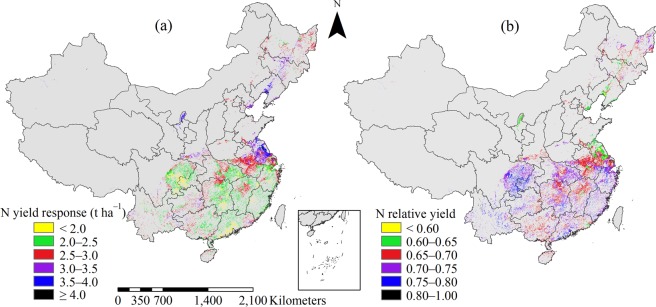
Distribution of yield response (**a**) and relative yield (**b**) to nitrogen (N) fertilizer application for rice in China.

On average, the N yield response was 2.7 t ha^−1^ across all sites, with 39.2% variation. The coefficients of variation (CVs) of N yield response were 37.3%, 36.8%, 37.9%, and 37.3% for early, middle, late, and single-season rice, respectively (Fig. 3a). The yield response in middle and single-season rice planting regions was higher than that in early and late rice planting regions, except for north SW. High N yield response (more than 3.0 t ha^−1^) was mostly observed in northeast MLYR, central NW and NE regions, which accounted for 18.7% of the study area. Low N yield response of <2.5 t ha^−1^ accounted for 47.1% of the study area and was mainly distributed in double-season rice planting regions and in the north SW. High N yield response means low N relative yield; sites with N relative yield of <0.7 were mostly located in the NE region, central NW, and northeast MLYR, with N relative yield values of <0.65 for 9.2% of sites in the study area (Fig. 3b). In the current study, the average N relative yield was 0.70 with 13.7% variation. The majority of N relative yield values were between 0.65 and 0.75, which accounted for 78.6% of the study area. The N relative yield was above 0.75 for about 12.2% of sites in the study area and was mainly located in the SW and SC regions.

**Figure 4:**
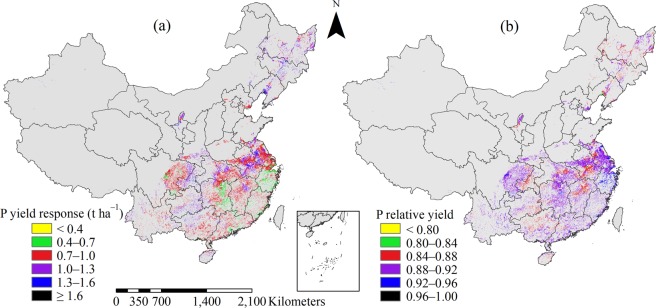
Distribution of yield response (**a**) and relative yield (**b**) to phosphorus (P) fertilizer application for rice in China.

The average P yield response was 0.9 t ha^−1^ across all sites, with 57.0% variation, and 1.0, 0.9, 0.7, and 1.0 t ha^−1^ for early, middle, late, and single-season rice, with 56.9%, 53.6%, 62.7% and 54.5% CVs, respectively. Sites with P yield response values of between 0.7 and 1.3 t ha^−1^ accounted for 85.8% of the study area (Fig. 4a). Sites with low P yield response (<0.7 t ha^−1^) accounted for 11.1% of the study area, and were mainly distributed in double-season rice planting regions, such as south and east MLYR and northeast SC regions. Sites with high P yield response of >1.3 t ha^−1^ were mainly located in the single-season rice planting regions. Sites with P relative yield values of between 0.88 and 0.92 accounted for 71.2% of the study area (Fig. 4b). Sites with P relative yield values of >0.92 accounted for 7.1% of the study area and were mainly located in east MLYR region. Sites with P relative yield of <0.88 accounted for 21.7% of the study area and were mostly located in the middle and single-season rice planting regions.

**Figure 5:**
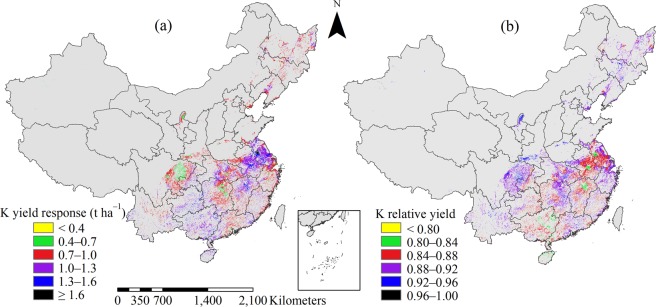
Distribution of yield response (**a**) and relative yield (**b**) to potassium (K) fertilizer application for rice in China.

The average K yield response was 1.0 t ha^−1^ across all sites with 53.4% variation; and 87.6% of sites in the study area had K yield response values in the range of 0.7–1.3 t ha^−1^ (Fig. 5a). The K yield response values for early, middle, late and single-season rice were similar at 1.0, 1.1, 0.9, and 0.9 t ha^−1^, with CVs of 54.1%, 51.1%, 53.3%, and 54.3%, respectively. Higher values of K yield response (>1 t ha^−1^) were mainly located in the northeast MLYR and southeast SW, which corresponded to the lower K relative yield in these regions. Sites with K relative yield values of <0.84 accounted for 4.0% of the study area (Fig. 5b). Sites with K relative yield values of 0.84–0.92 accounted for 88.7% of the study area. Sites with K relative yield of >0.92 accounted for 7.3% of the study area and were mainly located in the middle and single-season rice planting regions, such as central NW, central MLYR and north SW.

### Spatial distribution of nutrient requirements

**Figure 6:**
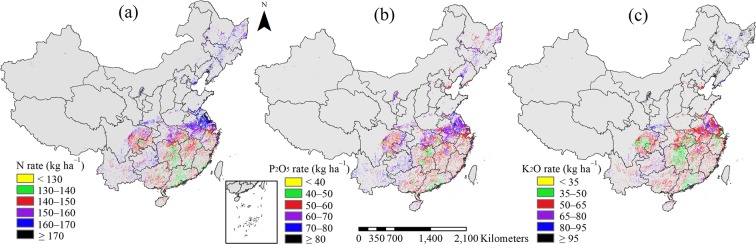
Distribution of fertilizer N (**a**), P_2_O_5_ (**b**) and K_2_O (**c**) requirements for rice in China.

Nitrogen fertilizer requirement showed an obvious spatial distribution between regions, and middle and single-season rice had higher N fertilizer requirement than early and late rice (Fig. 6a). Of the study areas, the sites of 20.9% had N fertilizer requirements higher than 160 kg N ha^−1^, mainly distributed in the NE, north MLYR and NW regions; the high N (>180 kg N ha^−1^) was needed for achieving high yield in some regions because of high attainable yield and N yield response (Figs 2a and 3a). Fertilizer N requirements of 140–160 kg ha^−1^ accounted for 66.4% of the study area, mainly located in the MLYR and SW, and north NE. In some early-late rice planting regions, a low N rate (130–140 kg ha^−1^ or less) in each season could meet the crop requirement, for example south MLYR and middle SC, which accounted for 12.8% of the study area.

There was strong spatial variability in P fertilizer requirement among regions, with a CV of 25.1% (Fig. 6b), and the CVs of P fertilizer requirement were 27.6%, 22.4%, 25.1%, and 23.2% for early, middle, late, and single-season rice, respectively. Most of the study areas (85.5%) were within the range of 50–70 kg P_2_O_5_ ha^−1^, mainly in the MLYR, SC, north SW, and north NE regions. In some middle and single-season rice planting regions, such as southeast SW, central NE, and north MLYR, 70 kg P_2_O_5_ ha^−1^ or more was needed to meet the crop requirement, which accounted for 6.7% of sites in the study area. For the remaining 7.8% of the study area, 50 kg P_2_O_5_ ha^−1^ was enough; these sites were mainly distributed in the early-late rice planting regions, such as south MLYR and south SC regions.

There was large variation in K fertilizer requirement among regions, with a CV of 38.8% (Fig. 6c). The CVs of K fertilizer requirement were 35.4%, 31.4%, 32.9%, and 20.2% for early, middle, late, and single-season rice, respectively. The K fertilizer requirements in middle and single-season rice were higher than for early and late rice, especially in NE and NW regions, with 12.5% of sites in the study area requiring >80 kg K_2_O ha^−1^. The average K fertilizer requirements needed to maintain the soil K balance in single-season rice planting regions were 99 kg K_2_O ha^−1^. In the SW region, the K fertilizer requirement was mainly within the range of 65–80 kg K_2_O ha^−1^, except in the north SW. In 73% of sites in the study area, the K fertilizer application rates were <65 kg K_2_O ha^−1^; these sites were mainly in the MLYR and SC, and north SW regions.

## Discussion

The rice yield has greatly increased worldwide in the past decades because of a series of agricultural practices, such as improved varieties, soil fertility management, and water, weed, pest and disease management^4,6^. The worldwide rice production could still be increased by 47% if 100% of attainable yield could be achieved^24^. China accounts for more than one fifth of world rice production mainly because of improved productivity per hectare; the unit yield increased by 1 t ha^−1^ in the past two decades^1^. However, the decreasing rate of rice yield increase is largely interpreted as a decreasing relative contribution of fertilizer^25^, and concerns have been raised about the synergetic development of sustainable rice production and environmental pollution^3,26^. There was large spatial variability in attainable yield due to differences in rice varieties, plant type of season and soil fertility among regions, and multiple-year/site experiments were collected to attain reasonable target yield when estimating nutrient requirements. In the current study, the CV of attainable yield was 18.3%, and the average middle and single-season rice yields were higher than those of early and late rice (Fig. 2), the one of reasons is because longer growth duration (20–30 more days) and larger day/night temperature differences contributing to higher dry matter accumulation in middle and single-season rice than in early and late rice.

Excessive and imbalanced fertilizer application has led to very serious environmental problems, such as runoff, water eutrophication, and greenhouse gas emissions^2,3^. Integrated technologies and fertilizer recommendation methods should be more carefully selected and deployed to continue to increase food production and avoid negative effects on the environment^11^, such as integrative crop management including optimize nutrient input at different growth-stage, plant density and irrigation management, which increased grain yield and recovery efficiency by 10% and 20%, respectively^27^. Multi-split topdressing, controlled-release N fertilizer application, and integration of water and fertilizer management can all significantly enhance grain yield and N uptake^7,28,29^. However, a reasonable parameter is necessary to express soil nutrient supply capacity to obtain a reasonable amount of fertilizer, because there are challenges in terms of a shortage of agricultural workers and high soil testing costs.

Fertilizer nutrients applied into soils will be eventually absorbed by plants and can be reflected by crop yield increase. The yield response to nutrient application is an important parameter to express soil nutrient supply capacity, and the 25th percentile, median, and 75th percentile of relative yield are used as coefficients to estimate yield response when yield response data for a particular field are not available^16^, and represent soil indigenous supply classes of ‘low’, ‘average’, and ‘high’, respectively. High yield response corresponds to low relative yield and low soil nutrient supply capacity, and means more fertilizer was needed. The relative yield values to soil indigenous supply classes were determined by different databases according to rice plant types (early, middle, late and single-season rice) in the *Nutrient Expert for Rice* system. The differences in attainable yield and yield response resulted in the variation of fertilizer requirements between fields. The large CVs of the yield response in the current study indicated that specific fertilizer recommendations should be conducted according to the differences in climate, yield levels, and nutrient uptake^21^. Understanding and analyzing spatial heterogeneity of yield response and relative yield will help to accurately determine fertilizer application rate.

Many studies have suggested that high yield can be attained under low fertilizer application rate or optimal nutrient management practices when compared with farmers’ standard practices^7,12,30^. In the current study, high fertilizer application rates were mainly located in NE, north MLYR and central NW, in which the high N and P fertilizer application rates were mainly related to high yield, high yield response and low relative yield. High K fertilizer application rates in the single-season rice planting regions were mainly because straw was not returned to the fields, as 84% of K is concentrated in the straw^21^. If the straw was returned into the soil for single-season rice planting regions, K fertilizer application rates could be reduced by 30% using *Nutrient Expert for Rice*. In addition, the residual nutrient from previous season also was considered to determine fertilizer requirements in the *Nutrient Expert for Rice* system.

With the continuous dynamic optimization of the *Nutrient Expert for Rice* system for each cropping season, the method increased grain yield and N recovery efficiency^16^. The policy support from the government plays a crucial role to control fertilizer consumption, such as “National Key Research and Development Program” was implemented from 2016 to develop high-efficient fertilizer recommendation method and establish nutrient-limits standard, in order to achieve “zero-growth” by the year of 2020. The fertilizer recommendation system combined with geographical information systems provides useful information for making agricultural policies or strategies, obtaining reasonable fertilizer distribution and supply, and ensuring food security^31^. These methods require holistic field information, including the spatial distribution of grain yield, soil nutrient supply capacity and nutrient requirements, so as to achieve sustainable agricultural development. Traditional training approach has a limited role in reducing farmers’ fertilizer use^32^, integrated technologies, substantial investment, policy interventions and formulation of legislation should be conducive in field-guidance to resolve the contradiction between food security and environmental protection in agricultural production systems.

## Conclusions

Chinese agricultural research must keep pace with the growing demands for food, high-efficiency utilization of resources and environmental protection. Coupling geographical information systems and fertilizer recommendation systems based on strong agronomic foundations is a sound approach, to provide actionable guidance in developing agricultural strategies and policies. In the current study, the spatial distribution of grain yield, yield response to nutrient application and relative yield were analyzed using geographical information systems based on a large number of field experiments; the nutrient requirements at regional scale were mapped and combined with the *Nutrient Expert for Rice* decision support system across major rice producing regions. We found great spatial variations in grain yield, yield response and relative yield between and within regions. The CVs of yield response to N, P, and K fertilizer application reached 39.2%, 57.0%, and 53.4%, respectively, across all study regions, which resulted in the spatial variation of nutrient requirements. The results of this study will help to manage natural resources, increase nutrient efficiency and productivity, and minimize environmental risk at regional or national scale.
